# Optimización de flujos de trabajo y paneles de cribado para la detección de gammapatias monoclonales malignas

**DOI:** 10.1515/almed-2019-0028

**Published:** 2020-08-10

**Authors:** Raquel Oliveros Conejero, Pilar Pascual Usandizaga, Adolfo Garrido Chércoles

**Affiliations:** Institución Servicio de Análisis Clínicos Hospital Universitario Donostia, San Sebastián, Guipúzcoa, España

**Keywords:** algoritmo de cribado, carga de trabajo, mieloma múltiple

## Abstract

**Objetivos:**

El mieloma múltiple (MM) es una de las malignidades hematológicas con más retraso en el diagnóstico. Frecuentemente, llegan al laboratorio peticiones de técnicas de detección de MM sin sospecha específica generando mucha carga de trabajo y baja eficiencia. Objetivo: aumentar la eficiencia de aplicación de los protocolos de cribado del MM.

**Métodos:**

Recopilación de resultados de electroforesis (EPS) e inmunofijación en suero (IFS) y orina (IFO), y de cadenas ligeras libres en suero (CLLS) de 75 pacientes de MM al diagnóstico, 3 amiloidosis y un plasmocitoma solitario. Recopilamos las alteraciones analíticas presentes en estos pacientes vs población control (n.=120). Validación del algoritmo de cribado en 261 pacientes consecutivos con sospecha clínica o analítica de MM.

**Resultados:**

Algoritmos de cribado más sensibles: EPS + CLLS o IFS + CLLS (98% de sensibilidad). Prospectivamente, el protocolo EPS + CLLS detectó 27 de las 28 gammapatia monoclonal (GM) diagnosticadas y ahorró 15 h laborales. Frecuencia de resultados analíticos alterados, 5 de los 6 parámetros eran más frecuentes en el grupo a estudio, acumulando ≥3 parámetros alterados el 61,1% vs. el 1,7% de la población control (Valor predictivo positivo: 85%; Valor predictivo negativo: 94%).

**Conclusiones:**

El protocolo de cribado EPS + CLLS fue el más sensible y menos laborioso. Además, permitió mejorar la sensibilidad diagnóstica y la eficiencia de trabajo.

## Introducción

El mieloma múltiple (MM), es un trastorno clonal de las células plasmáticas, con plasmocitosis de la médula ósea y, en la mayoría de los casos, producción de una inmunoglobulina monoclonal anormal detectable en la electroforesis de proteínas en suero (EPS) y/o orina. Las células plasmáticas neoplásicas y la producción de inmunoglobulina monoclonal causan daño en órganos y tejidos, dando como resultado las características clínicas típicas de MM, incluyendo lesiones óseas (que se manifiestan por dolor óseo, fracturas patológicas, osteólisis, entre otros), anemia, hipercalcemia y daño renal. Se han propuesto directrices nacionales e internacionales para guiar el diagnóstico y el seguimiento del mieloma múltiple [1], [2], [3].

La EPS es la técnica más utilizada para la detección de gammapatias monoclonales, sin embargo, esta prueba por si sola tiene sensibilidad limitada, principalmente cuando el componente monoclonal es pequeño o de cadenas ligeras libres únicamente [4]. Es por este motivo que, ante la sospecha de una gammapatia monoclonal (GM), el Grupo Internacional de Trabajo sobre el Mieloma (IMWG, por sus siglas en inglés) recomienda el uso de un conjunto de pruebas séricas para el cribado de la proteína monoclonal (PM): la EPS, la determinación de las cadenas ligeras libres en suero (CLLS), y la inmunofijación (IFS) [1]. Este algoritmo de cribado ofrece una elevada sensibilidad y especificidad diagnóstica, con la simplicidad de recurrir únicamente a la muestra de suero y la posibilidad de ahorrar al paciente y al laboratorio la recogida y manejo de una muestra de orina de 24 h. No obstante la sencillez del algoritmo recomendado, la realidad en nuestro centro es que se solicitan un alto número de proteinogramas en suero y orina sin que haya una indicación específica de sospecha de una GM, por lo que resulta difícil decidir cuándo aplicar el cribado recomendado por el IMWG. Sin embargo, la utilización de únicamente una prueba de cribado puede generar falsos negativos y por lo tanto retrasos en el diagnóstico con impacto en el tiempo de supervivencia del paciente y/o en comorbilidades más severas [5].

Teniendo como base esta problemática, se ha buscado con este trabajo lograr un objetivo doble: por un lado definir un perfil de sospecha de GM en base a i) la analítica (anemia, hipercalcemia, hiperproteinemia, aumento de creatinina, inmunoparesia), ii) la sintomatología del tipo dolor óseo y iii) la morfología sospechosa en el proteinograma, y por otro, definir el conjunto de técnicas que resultan más coste-eficientes para el cribado evitando de esta manera aplicar un panel innecesariamente complejo y de respuesta más tardía.

## Materiales y métodos

### Estudio retrospectivo

Se revisaron retrospectivamente desde el programa del laboratorio (OMEGA 3000) y de la Historia Clínica (Clinic), 79 pacientes diagnosticados entre 2011 y 2014 de los cuales 75 fueron de Mieloma Múltiple (MM), 3 de Amiloidosis (AL) y 1 de plasmocitoma solitario (PS); además se revisaron 120 pacientes control sin GM. Se recogieron los datos obtenidos dentro de los 30 días del diagnóstico de MM/AL/PS, antes de comenzar el tratamiento, para las pruebas: EPS, inmunofijación en suero (IFS) y en orina (IFO), y CLLS y en ambos grupos los siguientes signos de sospecha de mieloma: calcio sérico (Ca) > 10,2 mg/dL, valores de referencia (VR): 8,8–10,2 mg/dL, creatinina (Cr) > 1,8 mg/dL (VR: 0,4–1) o MDRD-4 < 60, anemia (Hb < 12 g/dL en mujeres y Hb < 13 g/dL en hombres), inmunoparesia (IP) (IgG < 7 g/L, VR: 7–16; IgA < 0,7 g/L, VR: 0,7–4; IgM < 0,4 g/L, VR: 0,4–2,3), hiperproteinemia (PT) > 8,7 g/dL (VR: 6,6–8,7) y afectación ósea. Se elige este periodo de 30 días para garantizar que los resultados de todas las pruebas normalmente realizadas durante el proceso del diagnóstico diferencial, estén disponibles.

Los valores de referencia adoptados son los aportados por los respectivos fabricantes.

### Estudio prospectivo

Se recogieron durante 1 mes (octubre de 2016) los mismos datos que en el estudio retrospectivo (Ca, Hb, PT, Cr, MDRD-4, inmunoparesia y dolor óseo) de todos los pacientes con solicitudes de su médico que incluyesen sospecha de GM o cualquiera de las siguientes pruebas: EPS, IFS, o IFO.

### Métodos

Para la EPS se utilizó el instrumento Capilarys 2 y para la IFS e IFO, el Hydrasys, ambos de Sebia, con los antisueros suministrados por la propia casa comercial. Las CLLS fueron determinadas en un SPAplus con el reactivo Freelite de The Binding Site, adoptando el intervalo de normalidad establecido para el cociente Kappa/Lambda libre de 0,26 a 1,65 [6]. En los pacientes con una disminución de la tasa de filtración glomerular (estimado a través de la fórmula de Modification of Diet in Renal Disease), y definida por un valor de MDRD <40 se ha considerado como intervalo de normalidad para el cociente kappa/lambda los valores de 0,37 a 3,1 ya que aporta mayor especificidad al ensayo [7].

Las determinaciones de bioquímica general (Calcio, Proteínas Totales, Creatinina, e Inmunoglobulinas) se realizaron en un COBAS c702 y la de hemoglobina en un SYSMEX XN, ambos de ROCHE.

### Cálculo del tiempo de dedicación por técnica

Para cada técnica utilizada en una sospecha de GM se buscó la siguiente información:

1. Tiempo hasta informar resultado final.
2. Tiempo que cada técnica requiere de dedicación exclusiva.
3. Tiempo de validación del resultado obtenido por cada técnica.

### Análisis estadístico

Se estudió la sensibilidad de cada una de las pruebas realizadas para la detección de la GM maligna (EPS, IFS, IFO, CLLS) y de las diferentes combinaciones para ver con cual se obtendría mayor sensibilidad diagnóstica.

Se calculó el valor predictivo positivo (VPP) de cada una de las alteraciones bioquímicas y el número de alteraciones para los que se aprecia diferencias significativas con respecto al grupo control.

El análisis de asociación de variables categóricas, se estudió a través de la prueba exacta de Fisher, así como VPP y valor predictivo negativo (VPN). Como dato de precisión se aportan los intervalos de confianza (IC) al 95%. Los análisis de datos se realizaron con los software MedCalc 10 y Excel.

## Resultados

### Análisis retrospectivo del estudio de PM: sensibilidad diagnóstica

Para definir un perfil de sospecha de GM integrando los datos analíticos y clínicos, se ha recurrido a la revisión de los datos de 79 pacientes con diagnóstico confirmado de MM o Amiloidosis primaria (AL) o plasmocitoma solitario (grupo de estudio) y de 120 pacientes sin diagnóstico de GM (grupo control). En 54 de los 79 pacientes del grupo de estudio, se disponía de los resultados de las 4 pruebas de detección de PM en el momento del diagnóstico: EPS, IFS, CLLS, IFO, y de las señales analíticas de sospecha listadas en el apartado material y métodos, en el momento del diagnóstico. Estos valores se usaron para estudiar la sensibilidad de cada una de las técnicas y de su uso combinado para la identificación de la PM (Tabla 1). Los 25 restantes se excluyeron del análisis porque no permitían hacer el estudio comparativo de sensibilidad diagnóstica.

**Tabla 1:** Sensibilidad de las diferentes agrupaciones de pruebas de laboratorio para la identificación de proteína monoclonal en pacientes diagnosticados con MM (52), AL (1), o Plasmacitoma (1).

Sensibilidad, %				
Perfil^a^	Sole	i EPS	IFS	IFO
CLLS+	91	98	98	91
EPS+	80		93	94
IFS+	93			94
IFO+	78			
EPS + CLLS+			98	98
EPS + IFS+				94

^a^CLLS, Cadenas ligeras libres en suero; EPS, electroforesis de protefnas en suero; IFS, inmunofijación en suero; IFO, inmunofijación en orina.

En este grupo de estudio, las pruebas más sensibles fueron la IFS y las CLLS, mientras que la EPS o el estudio de proteinuria de Bence Jones por IFO fueron las menos sensibles. Cuando se combinan varias técnicas se mejora la sensibilidad de identificación de PM hasta un máximo de 98%. Esta sensibilidad se obtuvo únicamente cuando se combinaron los resultados de CLLS con cualquiera de las técnicas en suero. En la ausencia del resultado de CLLS la sensibilidad máxima lograda no supera el 94%, incluso con la inclusión de la IFO. El único caso no identificado por el protocolo basado en suero corresponde a un paciente con plasmocitoma no-secretor y por lo tanto no identificado por ninguna de las 4 técnicas.

### Análisis retrospectivo del estudio de PM: perfiles de indicación de sospecha de GM maligna

El análisis retrospectivo de todos los casos de GM confirmada y de los controles (pacientes sin evidencias de una GM) permitió evaluar la incidencia de señales analíticas en cada uno de los grupos para identificar cual o cuales podrían, más específicamente, sugerir la presencia de una GM, o sea, generar una sospecha justificativa de cribado de PM. A los valores de calcio, creatinina en suero, inmunoglobulinas totales (IgG, IgA o IgM), hemoglobina, y proteínas totales en suero, se ha añadido la afectación ósea en el momento de presentación del paciente por ser descrito como el síntoma CRAB (acrónimo inglés para hipercalcemia C, afectación renal R, anemia A, afectación ósea B) más frecuente en el diagnóstico del MM [8].

Se ha observado que, con excepción de la hipercalcemia y la afectación renal, todos los parámetros analizados se encuentran fuera de sus valores de referencia (descritos en Material y Métodos) con mayor frecuencia (p<0,05) en la población de pacientes diagnosticados de GM maligna que en la población control (Tabla 2). Sin embargo, solamente las PT > 8,7 g/dL y la afectación ósea presentan un valor predictivo positivo (VPP) superior al 80%: 100% (IC 95% = 82–100%) y 85% (IC 95% = 66–96%) respectivamente. El 43,3% de los controles presentaron al menos una de las señales o uno de los síntomas estudiados, pero solamente un 1,7% acumula 3 o más variables alteradas a la vez. En cambio, en la población con diagnóstico de MM, el porcentaje de pacientes con tres o más variables alteradas asciende a un 61%.

**Tabla 2:** Frecuencia de alteraciones de las variables estudiadas en las poblaciones control y patológica.

	Control 120, n (%)	MM/AL 54, n (%)	VPP%	VPN%, (IC 95%)	S%	E%
(A) Variable alterada						
Hiperproteinemia	0 (0)	19 (35)	100 (82–100)	77 (70–84)	35 (23–49)	100 (97–100)
Hipercalcemia	9 (7)	9 (16)	50 (26–74)	71 (63–78)	17 (8–29)	92 (86–96)
Anemia	27 (22)	41 (76)	60 (48–72)	88 (80–93)	76 (62–86)	77 (69–85)
Deterioro funcion renal	11 (9)	12 (22)	52 (31–73)	72 (64–79)	22 (12–36)	91 (84–95)
Inmunoparesia	18 (15)	43 (79)	70 (57–81)	90 (83–95)	79 (66–89)	85 (77–91)
Afectacion osea	4 (3)	23 (43)	85 (66–96)	78 (71–85)	43 (29–57)	97 (92–99)
(B) Variables alteradas por paciente^a^						
0	68 (57)	0(0)				
≥1	52 (43)	54 (100)	49 (41–61)	100 (95–100)		
≥2	15 (12)	43 (80)	74 (61–85)	90 (84–95)	80 (66–89)	87 (80–93)
≥3	2 (2)	33 (61)	94 (81–99)	85 (78–90)	61 (47–74)	98 (94–100)

n, № pacientes; VPP, valor predictivo positivo; VPN, valor predictivo negativo; S, sensibilidad; E, especificidad. ^a^ Se describe el porcentaje de pacientes que presenta ninguna variable alterada, al menos 1, 2, three o más variables alteradas a la vez. El VPP y el VPN se calcularon para cada subgrupo frente al total de pacientes.

### Cálculo del tiempo de trabajo para cada técnica de estudio de PM

Revisando los datos históricos de nuestro laboratorio se observa que, de media, cada mes se piden unas 3,180 EPS, 343 IFS, y 442 IFO. Se cuenta con cuatro equipos para realizar estas técnicas. Se ha estimado el tiempo aproximado que tarda cada una de las pruebas en realizarse/validarse:

1. IFS = 75 min para una placa de 4 muestras (con dilución, aplicación, migración y tinción)
2. IFO = 75 min para una placa de 9 muestras (con dilución, aplicación, migración y tinción)
3. Electroforesis = 80 muestras/hora (h)
4. Validación de pruebas = 45/hora

Considerando 22 días hábiles en un mes, se obtienen los siguientes tiempos medios de dedicación diaria:

1. IFS = 4,9 h/día (343 peticiones/4 muestras por placa × 75 min por placa/22 días)
2. IFO = 2,79 h/día (442 peticiones/9 muestras por placa × 75 min por placa/22 días)
3. EPS = 1,8 h/día (3,180/80 muestras por hora)/22 días)
4. Validación de pruebas = 4 h/día (3,965 pruebas/45 pruebas por hora/22 días)

Se estima un tiempo total de realización de estas pruebas de 13,49 h/día (suma de los puntos 1 a 4)

Se observa que, un 20% de las EPS, un 50% de las IFS y un 25% de las IFO solicitadas corresponden a pacientes nuevos, o sea, sin previo diagnóstico de GM. Por lo tanto, el estudio de nuevos pacientes podría representar un 31,5% del tiempo de trabajo debido a la realización de estas técnicas de laboratorio. El porcentaje de resultados negativos observado en los pacientes nuevos fue un 88% para la EPS, un 73% para la IFS y 93% para la IFO.

Estudio prospectivo de la eficiencia del algoritmo de cribado EPS + CLLS: sensibilidad y tiempo de trabajo.

**Figura 1:**
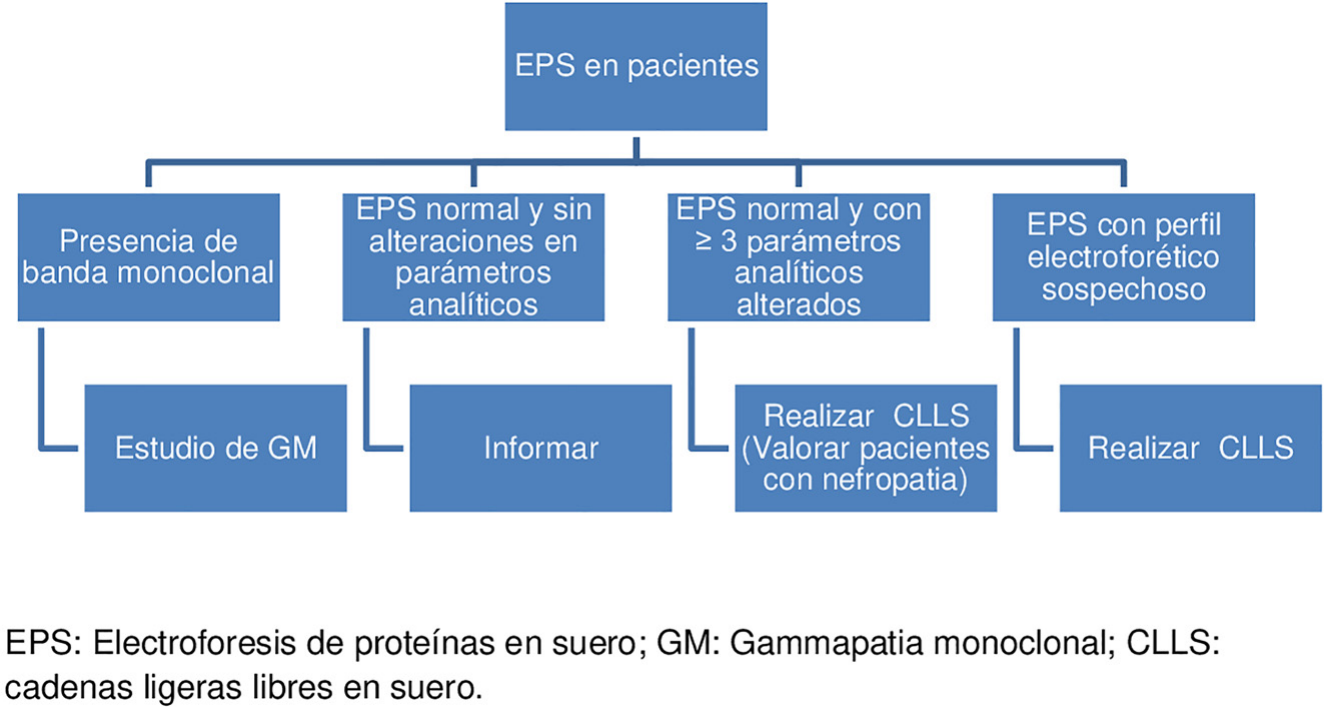
Algoritmo propuesto para el cribado de gammapatia monoclonal.

Se seleccionaron prospectivamente durante 1 mes, los pacientes consecutivos con petición de EPS y 2 o más señales de sospecha (Tabla 2), o de una IFO, o IFS, sin previo diagnóstico de GM, siguiendo el algoritmo propuesto en la Figura 1. De los 261 pacientes un 48% fue por petición de EPS únicamente y un 3% por petición de IFO. Los demás pacientes incluidos resultaron de peticiones que incluían la combinación de más de una técnica de laboratorio (Tabla 3). Al cabo de seis meses, se volvió a revisar la clínica de estos pacientes para verificar si la sospecha inicial de GM se mantenía y se confirmaron 28 diagnósticos.

**Tabla 3:** Tipo y frecuencia de perfiles de pruebas relacionados con la detección de componentes monoclonales.

Perfil^a^	Solicitudes, %^b^
Solo IFO	8 (3%)
IFO + EPS	42 (16%)
IFO + EPS + IFS	18 (7%)
Solo EPS	125 (48%)
EPS + IFS	68 (26%)
Total	261 (100%)

^a^IFO, Imnunofijacion en orina; EPS, Electroforesis de protelnas en suero; IFS, lnmunofijacion en suero. ^b^Frecuencia de cada tipo de peticion recibida en el laboratorio.

Del estudio retrospectivo, se observó que la aplicación conjunta de la EPS y CLLS resultaba el protocolo más sencillo y a la vez sensible para la detección de nuevas proteínas monoclonales. Aplicando este algoritmo de manera prospectiva al grupo de 261 pacientes observamos que, con la excepción de una GMSI, se identifican todas las GM diagnosticadas (27 de 28). Con el algoritmo propuesto, disminuiría la realización de IFS en un 68% y de IFO en un 60% (Tabla 4). O sea, se realizarían solamente en situaciones donde el protocolo de cribado EPS + CLLS fuese positivo o se tuviese sospecha de AL. La aplicación del nuevo protocolo podría suponer una disminución del tiempo de trabajo en 15 h (considerando 50 min para analizar 20 muestras de suero con el ensayo CLLS y 4 minutos para su validación).

**Tabla 4:** Impacto de la aplicación del nuevo protocolo de cribado basado en EPS + FLC.

Prueba diagnostica	№ peticiones recibidas^a^	∆№ pruebas con nuevo protocolo (EPS + FLC)^b^	∆tiempo (h) de ejecucion con nuevo protocolo	∆tiempo (h) de validacion con nuevo protocolo
IFO	68	−40	−6,4	−0,9
IFS	86	−58	−19,3	−1,4
EPS	253	+8	+0,3	+0,2
FLC	0	+261	+10,9	+1,0
Total	407	+171	−14,5	−1,1

^a^Número de peticiones en las que se solicitaba al laboratorio cada una de las pruebas en el periodo del estudio prospective. Este numero es superior al de pacientes debido a que en el 49% de ellos se solicito mas de una prueba. ^b^El cálculo para la IFO e IFS se basó en que solamente las muestras de los 261 pacientes con diagnosticos positivos de GM (28) o con sospecha de AL (0) necesitarian realizar la respectiva prueba; La EPS y las FLC se aplicarian a todos los pacientes estudiados (261).

## Discusión

El diagnóstico de una GM maligna suele tardar unos seis meses desde que el paciente acude por primera vez a su médico, debido a la sintomatología, hasta que se produce el diagnóstico final [9]. Esto se debe, por un lado, a que los síntomas son muy inespecíficos y, por otro, a un bajo índice de sospecha relacionado con la rareza relativa de la enfermedad. Esta realidad origina, frecuentemente, procesos de cribado poco eficientes y elevadas cargas de trabajo para el laboratorio. Con el doble objetivo de mejorar el proceso de cribado y aumentar el índice de sospecha se ha buscado con este trabajo:

1. Identificar el conjunto de técnicas con la máxima sensibilidad y menor carga de trabajo.
2. Identificar un perfil de señales analíticas de sospecha que permitiera generar un algoritmo de cribado de GM con mayor especificidad.

En línea con otros autores, [4], [5], [6], [7], [8], [9], [10] este trabajo demuestra la sensibilidad del panel de cribado de GM sintomáticas constituido por la EPS y las CLLS en suero. Sin embargo, por sí solo, la aplicación del panel no conlleva a una mejora significativa de la eficiencia de todo el proceso de cribado. Es necesario saber en qué situaciones se justifica su aplicación, principalmente cuando no exista por parte del clínico una sospecha específica. Con este trabajo hemos identificado que la alteración de 3 o más parámetros analíticos en un mismo paciente debería generar una sospecha de GM y la aplicación del panel de cribado (EPS + CLLS) ya que es un hecho significativamente menos probable de ocurrir en pacientes sin MM (<2%), que en pacientes diagnosticados con MM o AL (61%). Desconocemos otros estudios que hayan realizado este tipo de aproximación analítica, valorando la incidencia de señales simultáneas de sospecha en una población control frente a una población patológica. El trabajo de Goldschmidt y colaboradores [8] hace una aproximación semejante, pero elige la población control basado en dolor de espalda. Los autores concluyen que el dolor de espalda acompañado de: cansancio, pérdida de peso, o resultados de laboratorio anormales debería generar un alerta de MM. Desde el punto de vista del clínico es un algoritmo de sospecha útil, pero no tanto para el laboratorio debido a que la sintomatología del paciente, en concreto, el dolor de espalda, el cansancio y la pérdida de peso, no está fácilmente disponible. Otros trabajos han estudiado también la frecuencia de varias señales o síntomas, pero de manera individual y casi siempre en poblaciones patológicas [11, 12]. La limitación de este tipo de análisis viene del VPP pues no suele predecir de manera suficientemente específica la presencia de un MM. Esto se debe a que, a pesar del elevado porcentaje de pacientes con MM que se presentan con algunas de estas alteraciones, como anemia o disfunción renal, existen otro tipo de enfermedades mucho más frecuentes que comparten estos mismos síntomas. El dolor de espalda es un síntoma muy frecuente en los pacientes diagnosticados con MM, sin embargo, menos del 1% de la población que se presenta con este síntoma tiene una enfermedad maligna [13]. El valor de 80% para el VPP es comúnmente asumido como el umbral mínimo para considerar que una variable tiene suficiente poder predictivo para ser usado en la práctica diaria. En nuestro trabajo, a pesar de que la frecuencia de 5 de las 6 variables estudiadas ha sido significativamente superior en la población patológica, en línea con trabajos anteriores [8], solamente las PT > 8,7 g/dL y la afectación ósea presentaron VPP > 80%. Sin embargo, su frecuencia individual en la población patológica es inferior al 50%, lo que significa que el índice de sospecha de MM no se vería incrementado de manera significativa. Por este motivo se ha analizado la incidencia de varios síntomas a la vez en un mismo paciente para incrementar tanto el VPP como el VPN. La construcción de algoritmos de alarma basados en resultados analíticos más generales y que lleven a la realización de técnicas de cribado más específicas y sensibles, es cada vez más frecuente en los laboratorios de los hospitales. Estos algoritmos permiten mejorar la rapidez de diagnóstico, los flujos de trabajo y por tanto disminuir los desplazamientos de los pacientes a los centros clínicos.

Sin embargo, y a pesar de los resultados prometedores, hay que destacar que la proporción de pacientes del grupo de estudio frente a población control (54:120) utilizada en este estudio retrospectivo, es muy distinta a la que se verifica en la realidad, donde la prevalencia del MM es de 4–6 casos por cada 100.000 habitantes. Es una limitación de nuestro trabajo que aconseja prudencia en la interpretación de las cifras obtenidas para los VPP y VPN. A pesar de tal limitación, el análisis prospectivo de los 261 pacientes ha demostrado que la aplicación del algoritmo de cribado EPS + CLLS, en situaciones de sospecha clínica o analítica (basada en la combinación de síntomas) permite reducir la carga de trabajo de manera significativa y, a la vez, aumentar la sensibilidad diagnóstica frente a una única prueba (EPS o IFO). Este trabajo no pretende ser un estudio de modelo de economía de salud sino aportar aún mayor confianza a nuestros métodos de trabajo.
